# Development and Evaluation of a Loop-mediated Isothermal Amplification Assay for Rapid Detection of *Theileria annulata* Targeting the Cytochrome B Gene

**Published:** 2018

**Authors:** Melek CHAOUCH, Moez MHADHBI, Sassi LIMAM, Mohamed Aziz DARGHOUTH, Souha BENABDERRAZAK

**Affiliations:** 1. Laboratoire de Parasitologie Médicale, Biotechnologies et Biomolécules, Institut Pasteur de Tunis, Tunis, Tunisia; 2. Laboratoire de Parasitologie, Ecole Nationale de Médecine Vétérinaire de Sidi Thabet, Université de la Manouba, Manouba, Tunisia

**Keywords:** *Theileria annulata*, LAMP, Cytochrome b, Tunisia, Diagnosis

## Abstract

**Background:**

*Theileria annulata* is an economically important cattle disease in North Africa that occurs in subtropical and tropical areas. Accurate and rapid, molecular diagnosis of tropical theileriosis is an important issue that allows early treatment and, prevents transmission. We developed and validated a *Theileria annulata* specific LAMP assay targeting the cytochrome b multicopy gene, in order to increase the DNA detection sensitivity.

**Methods:**

The methodology was used to evaluate the occurrences of *T. annulata* in 88 field samples collected in Northern Tunisia during 2013–2014. The specificity and sensitivity of the LAMP assays were compared to conventional cytochrome b PCR and routine microscopy commonly used on naturally infected cattle blood samples.

**Results:**

The PCR assay showed a sensitivity of 70% and specificity around 75%. Our LAMP assay showed a suitable sensitivity 78.7% and specificity 87.5%, with, however, positive (98.4%) and negative (29.1%) predictive values.

**Conclusion:**

The LAMP assay is a simple and convenient diagnostic tool for tropical theileriosis. Moreover, LAMP does not require experienced staff and specialized equipment for sampling procedures and it is practical outside laboratories and can be used for field diagnosis.

## Introduction

Tropical theileriosis caused by the haemoprotozoan *Theileria annulata,* is an economically important protozoan disease affecting cattle in tropical and subtropical regions (1). *T. annulata* is transmitted by several species of ixodid ticks of the *Hyalomma* genus after their cyclical development (1). The disease is distributed over a wide geographical area, ranging from the Mediterranean littoral regions of Europe and Africa through the Middle East to India and China.

Tropical theileriosis which evolves in different subclinical forms affect both milk and meat production (2, 3).

Several methods for the diagnosis of *T. annulata* infection are available however they are difficult to use and not well suited for direct testing in the field, the difficulties are primarily encountered in the routine clinical diagnosis of theileriosis and in the direct microscopic detection of the parasites in tissue samples (4). These methodologies are used in the detection of acute cases but have limited value for the detection of carrier cases, where low numbers of erythrocytes are infected with piroplasms. The parasitological test is highly specific, but is time-consuming and is for its sensitivity dependent on the tissue sample, and the quality of the reading.

Several diagnostic assays including serological and PCR based assays have been developed for the detection of *T. annulata* infections. However, serological assays, such as the indirect fluorescence antibody test (IFAT), the indirect ELISA and the cELISA (5–7), does not allow the discrimination between infected and cured individuals nor the detection of asymptomatic cow with low antibodies levels.

The PCR-based assays including reverse line blotting (RLB) (8,9), while highly sensitive and specific (10–13) require experienced staff and specialized equipment making them difficult to use in the field.

Loop-mediated isothermal amplification (LAMP) is a simple technique that amplifies DNA with a high sensitivity and rapidity under isothermal conditions (14). This method combines the high sensitivity of a molecular diagnostic test to a simplicity that makes it suitable to field conditions with limited technical resources. Recently, loop-mediated isothermal amplification of DNA has been successfully developed for the detection of some *Theileria* species. It has targeted partial mRNA (15), UTRTu6 gene (16), PIM and p150 genes (17), p33 gene (18), 18S rRNA and ITS (19, 20).

However, targeting a multicopy gene may result in increased sensitivity of the LAMP assay (21). Indeed, compared to ribosomal DNA-based tests, a cytochrome b gene-based DNA amplification test is 20% more sensitive for the detection of piroplasms (21).

The aim of the present study was to characterize the reliability of a LAMP based assays targeting the cytochrome b gene, a parasite multicopy gene for the detection of *T. annulata* in cattle suspected of clinical theileriosis. The specificity and sensitivity of this LAMP assays were compared to routine microscopy and conventional PCR using blood samples from naturally infected cattle.

## Materials and Methods

### Collection of blood samples, Microscopy and DNA extraction

Overall, 88 field blood samples were collected from cattle suspected of clinical theileriosis from 2013 to 2014 in Northern Tunisia where piroplasmosis is enzootic. Sampling consisted of males and females (8 months to 5 yr) of different breeds (73 Crossbreds, 13 Friesians, 1 Brown Swiss and 1 local breed).

The study was approved by Ethics Committee of the university.

Cattles were examined for occurrences of visible clinical signs of the disease, such lymph node enlargement, fever, anorexia, and a rapid loss of condition. Blood samples collected in ethylenediamine tetra-acetic acid-containing tubes and were used to prepare thin blood smears and then stored at −20 °C until subsequent deoxyribonucleic acid (DNA) purification. Thin blood smears were fixed in methanol for 5 min, stained in Giemsa stain diluted at 10% with neutral distilled water for 20 min and examined under an oil immersion objective at a magnification of ×100 for the presence of intracellular forms with morphology compatible with *Theileria* (22). For this work, the detection of *T. annulata* parasites by microscopy was used as standard.

DNA was extracted from 300 μl of cattle blood using the Promega Wizard Genomic DNA Extraction Kit (Madison, WI, USA) following the manufacturer’s instructions. The extracted DNA suspended in 100 μl of the rehydration buffer and stored at −20 °C. These DNA samples were analyzed using both LAMP and PCR method.

### PCR amplification

The PCR primers were designed on the basis of the cytochrome b gene sequences of *T. annulata* available on Genbank and GeneDB. The selected primer pair amplifies a fragment length of 257 bp. PCR for cytochrome b (cyt b) was performed in a final volume of 25 μl containing 5 μl of purified DNA sample or control, 1.5 mM MgCl_2_, 0.2 μM of each deoxynucleotide, 1.25 U of Taq DNA polymerase and 50 pmol of each primer (TheilcytF+150/TheilcytR+407). Reactions were carried out in a TECHNE model TC512 using the following cycling conditions: an initial denaturation step at 94 °C for 5 min, 35 cycles at 94 °C for 1 min, 62 °C for 1 min and 72 °C for 1 min and a final elongation step at 72 °C for 10 min. Amplicons were electrophoresed in 1% agarose gels stained with ethidium bromide and visualized under UV light. Standard DNA fragments (100 bp ladder, Fermentas) were used to size PCR products.

### Design of Loop-mediated isothermal amplification (LAMP) primers

The LAMP primer sets were designed using Primer Explorer Ver.4 (http://primerexplorer.jp/e/), from the *T. annulata* sequence (XM_949625) of the cytochrome b mitochondrial multi copy gene available on Genbank. A set of four LAMP primers (Table 1) recognizing six specific sections of the *T. annulata* cytochrome b gene were designed. Two additional loop primers, loop forward (LF) and loop backward (23) were designed manually. The Loop primers were added to increase the number of loops in the reaction thereby increasing the speed of reaction (Table 1).

**Table 1:** List of specific LAMP and PCR primers for cytochrome b gene of *Theileria annulata* used in this study

***Primer***	***LAMP primer sequences***
BIP	5′ ATCACTCGTTTGGAGTTTCGTTTTAGGTAAATGATTACTAGAATACCACA 3′
FIP	5′ GAACAAACCAACCGAAACAAATGTAAAGGTATGGCTTTTGAAAGT 3′
F3	5′ CTTTCTTTTATGTGCCAGCA 3′
B3	5′ ACCAGAATACCAAGACCAA 3′
LF	5′ CCGAAACAAATGTTTAACAT 3′
LB	5′ TTTATGTTTCTACATATCAT 3′
	***PCR primer sequences***
TheilcytF	5′ GTATGGCTTTTGAAAGTACTTTGG 3′
TheilcytR	5′ TTCCGAAAAATTTCAATAAACCACCT 3′

### LAMP reaction

The *T. annulata* cytochrome b specific LAMP reaction was standardized for optimal temperature and time. Different parasites strains (*Babesia bovis*, *Babesia bigemina*, *Leishmania tropica*) were used as references to test LAMP specificity. Briefly, the LAMP assay was carried out in a 25 μl reaction mixture containing 10X Bst-DNA polymerase buffer (2.5 mM), betaine (10 μM), deoxynucleotidetriphosphates (2.5 mM), MgSO_4_ (10 mM), FIP and BIP (1.6 mM), loop-F and loop-B (0.8 μM), F3 and B3 primers (0.2 mM), Bst DNA polymerase (8U, New England Bio Labs), ddH_2_O and DNA template (1 μl).

The LAMP test was carried out for 45 min at 64 °C and ended by increasing the temperature to 80 °C for 5 min in a thermocycle TECHNE model TC512. LAMP product was subjected to electrophoresis in 1.5% agarose gels stained with ethidium bromide and was documented by UVP BioSpectrum AC Imaging System.

### Statistical analysis

First, we estimated sensitivity and specificity of PCR and LAMP in a 2×2 contingency table using Giemsa stained blood smears as the reference test and computed exact binomial 95% Confidence Intervals (CI). The sensitivity and specificity of tests were determined as follows (TP is True Positive, TN represents True Negative, FN is False negative and FP is False Positive): A: Sensitivity=TP/(TP+FN) ×100; B: Specificity=TN/(TN+FP) ×100, C: Positive predictive value=TP/(TP+FP) ×100, D: Negative predictive value=TN/(TN+FN) ×100. We compared the sensitivity and specificity of LAMP and PCR tests with the reference microscopic diagnosis. We performed also other statistical tests: Likelihood ratios (LR) and *Cohen’s kappa coefficient*. The LR is post-test measures that overcome the drawbacks of predictive values because they do not depend mathematically on prevalence, thus being more portable. Likelihood Ratios (LR) were calculated, taking LR for the positive results (LR+) and LR for the negative results (LR−). The degree of agreement between two tests was determined by *Cohen’s kappa coefficient* (ϰ) values with 95% confidence intervals. Kappa values express the agreement between two tests and a ϰ value of 0.21–0.60 represents fair to moderate agreement, ϰ > 0.60–0.80 represents substantial agreement, ϰ > 0.80 represents almost perfect agreement. Confidence intervals (CI) at 95% level for sensitivity, specificity, and overall accuracy were produced. The CI 95% is a range of values that has a 95% chance of containing the true value of the estimated parameter.

## Results

### Sensitivity and Specificity of the T. annulata specific LAMP

**Fig. 1:**
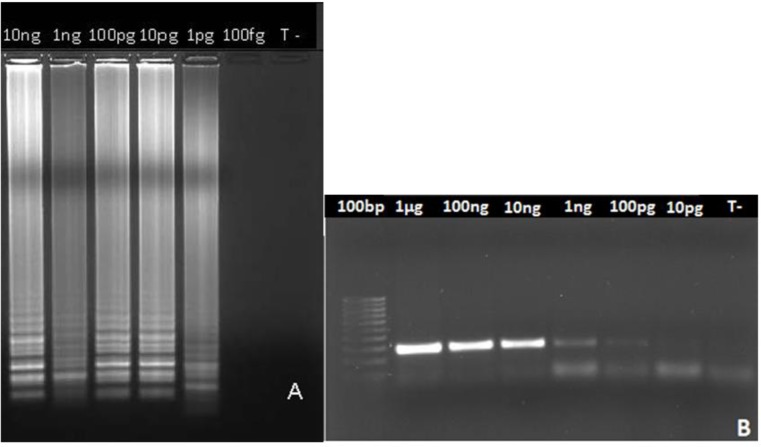
LAMP (A) and PCR (B) studied by agarose gel electrophoresis. Genome DNAs from 10-fold serially diluted *T. annulata* schizont-infected lymphocytes were used as templates

A set of oligonucleotide primers targeting *T. annulata* cyt b gene sequences were designed for the LAMP assay. To evaluate the sensitivity of LAMP, compared with PCR, DNA samples from serial dilutions of *T. annulata* schizont-infected lymphocytes were subjected to LAMP and PCR. While 100 pg of DNA was needed for conventional cyt b PCR to detect the parasite, our LAMP assay was able to detect DNA parasite from as low as 1 pg of *T. annulata* schizont-infected lymphocytes DNA suggesting that LAMP was 100 times more sensitive than PCR (Fig. 1). The specificity of the primer sets designed was first tested and confirmed in silico with cytochrome b gene sequences of different *Theileria* species as *T. parva* (Z23263.1), *Babesia equi* (XM_004833827) and *T. annulata* (KP731977) available in the DNA reference databases. Then, DNA samples from bovine *Babesia* species and *Leishmania* were used to evaluate the specificity in situ of our LAMP assay. Uninfected cattle blood and distilled water were used as controls. No cross-reactivity for our in-house primers that were unable to amplify the target gene in other pathogenic species. The negative samples also tested negative.

### Detection of field samples using microscopy, PCR, and LAMP assay

**Fig. 2:**
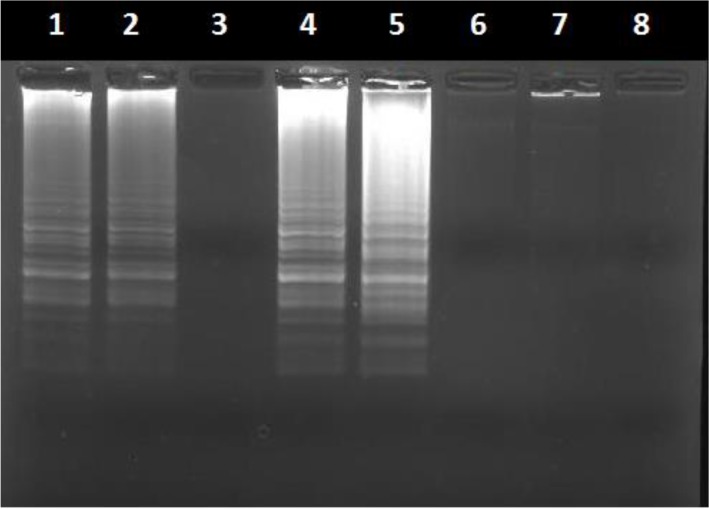
Amplification of different clinical DNA samples by loop-mediated isothermal amplification (LAMP) assays targeting the cytochrome b gene. (1) Positive control *T. annulata* DNA, (2-4-5) positives clinical samples, (3-6-7) Negatives clinical samples extracted from cattle blood, (8) H_2_O Negative control

**Fig. 3:**
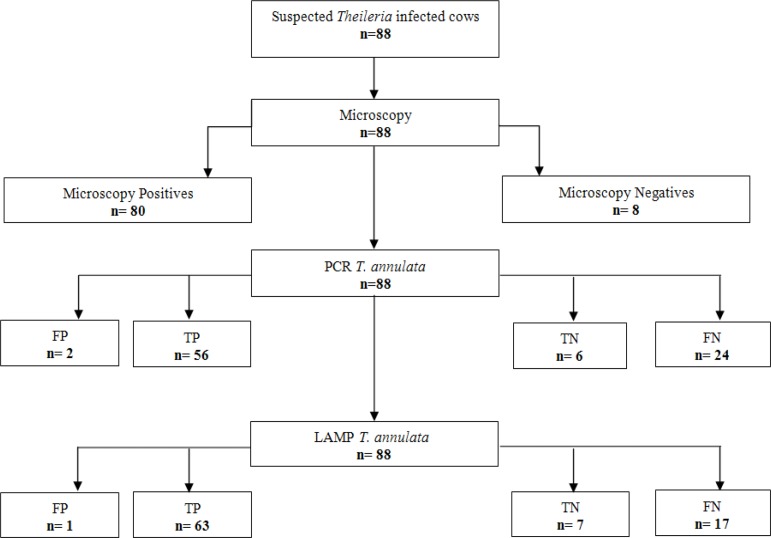
Flow-diagram of the results obtained from diagnostics techniques applied to clinical samples. (TP is True Positive, TN represents True Negative, FN is False Negative and FP is False Positive)

Parasitological examination by microscopy of the 88 collected samples revealed that 80 animals were infected with *Theileria*. The remaining 8 cows were microscopically negative and were thus considered as uninfected. The 88 biological samples were next evaluated with PCR and LAMP assays. *T. annulata* DNA was detected by LAMP in64 samples and by PCR in 58 samples (Fig. 2). However, the number of false positives was very low, which indicated a high specificity. The number of false negative indicates a higher sensitivity level for LAMP. The results obtained from the different diagnostic techniques are presented in the flow diagram (Fig. 3).

### Statistical analysis

The test performed on PCR, and LAMP showed a corresponding sensitivity of 70% and 78.7% respectively and a specificity of 75% and 87.5% respectively. The best positive likelihood ratio (LR+) was obtained for LAMP (6.3), followed by PCR (2.8). The best negative likelihood ratio (LR−) was achieved by LAMP with 0.24, then by PCR (0.4). The statistical evaluation of the LAMP, performed on 88 DNA extracted from cows’ blood samples obtained from Northern Tunisia region, gave a suitable sensitivity and a specificity fully in agreement with direct parasitological diagnosis. Our assay showed a good positive predictive value (PPV) and negative predictive values (NPV) (Table 2). Statistical analyses performed support the robustness of LAMP assay. LAMP assay obtained the best positive likelihood ratio (LR+ = 6.3). This value confirms that the animals diagnosed as positive with LAMP technique, are really positive. LAMP also showed negative likelihood ratio (LR−) greater than 0.1 (0.24) but lower than 1 which mean that the probability of a sample to give a negative result while it is positive is low. Subsequently, we measured the degree of agreement of the PCR and LAMP microscopy using *Cohen’s kappa coefficient*. Fair and moderate levels of agreement were recorded respectively for PCR (kappa=0.24) and LAMP (kappa= 0.34). Furthermore, the LAMP technique correlated better with the blood smear results since it gave the highest values of the *Cohen’s kappa coefficient*.

**Table 2:** Statistical analysis of LAMP and PCR based on cytochrome b gene by comparing with microscopy method for detection of *Theileria annulata* field samples (95% CI)

	***Microscopy***						
	**Sensitivity**	**Specificity**	**PPV**	**NPV**	**LR+**	**LR−**	**Kappa**
PCR	70 (58.7–79.7)	75 (35–96)	96.5 (88–99.4)	20 (7.7–38.5)	2.8 (0.8–9.3)	0.40 (0.2–0.6)	0.20 (0–0.4)
LAMP	78.7 (68.1–87.1)	87.5 (47.38–97.9)	98.4 (91.5–99.7)	29.1 (12.6–51)	6.3 (1–4)	0.24 (0.2–0.4)	0.35 (0.1–0.5)

Positive Predictive Value (PPV) / Negative Predictive Value (NPV) / Likelihood ratios (LR)

## Discussion

Tropical theileriosis has extensive prevalence and mortality rates with high impact in economy in several countries (24). The limited specificity of direct parasitological diagnosis led to the development and the use of more sensitive and specific diagnostic tools especially in cases of low parasitaemia, particularly in carrier cattle, to detect the parasite.

In this study, we describe the development of a rapid, simple and sensitive loop-mediated isothermal amplification (LAMP) assay for the species-specific detection of *T. annulata* in blood samples from cattle. The results are compared with conventional cyt b PCR and microscopy.

The detection of *Theileria* parasite in blood smears by microscopy is considered as a gold standard for the diagnosis of Theileriosis in our study. Indeed, despite its limited sensitivity (which may introduce a bias in the evaluation of the specificity of the other techniques used), this test, used in the clinical service of the veterinary units, is the only test that insure the effective presence of the parasite in blood of suspicious cows. The slides were examined at the National School of Veterinary Medicine of Sidi Thabet by two trained and experienced persons (a veterinarian and a technician). The microscopic diagnosis revealed 80 positive samples out of 88. We have next blind tested the DNA extracted from the 88 cows for the presence of *Theileria* parasites using our LAMP and PCR assays targeting cytochrome b gene.

Recently, different molecular tests targeting multi-copy genes have been developed for sensitive, specific detection of *T. annulata* in either the tick vector or bovine host (10, 13). Our choice was focused on cytochrome b gene as cytochrome b gene-based PCR presents a high level of sensitivity (25) certainly due to the multicopy nature of the mitochondrial gene. In addition, multiple mitochondria may be present in each cell, although the number per cell can vary widely between organism and tissue type (26). The PCR assay targeting the cyt b gene that we performed gave a lower sensitivity (70%) and specificity (75%) than LAMP (sensitivity 78.7% and specificity 87.5%). In our hands, however, both assays are less sensitive than the microscopic examination that revealed 80 positive samples out of the 88. This result is in disagreement with the one reported, besides the relevance of the cyt b based PCR for detecting carrying individuals in field samples show that the PCR assay provides greater sensitivity than the standard microscopic method (25).

Our results are however consistent with those reported in previous studies targeting the hypothetical protein (GeneDB TA04795), the 18S rRNA and the ITS genes (15, 19). The reliability of the results and especially the sensitivity can depend on various factors such as the presence of PCR inhibitors, DNA fragmentation, the successive passage of amplicons from one test tube to the other (carryover effect), the temperature profile of the reaction and the primer specificity.

Moreover, compared to these target genes, the multi-copy natures of the cyt b gene allows more reliability and represents an excellent target gene for the development of species-specific and sensitive LAMP assay (27).

Genetic differentiation was detectable between geographically separated populations of *T. annulata* (28). As a previous developed LAMP has been tested on Sudanese and Chinese strains, genetically separated from the Tunisian ones (30), their use in the Mediterranean region may bias the results. The LAMP that we have developed offers an alternative and could be used in the various countries including those of the Mediterranean region and would improve the diagnosis of tropical theileriosis. Although in cattle in Tunisia there are only *T. annulata* species, LAMP technique specificity must be confirmed on a larger number of control samples like *T. lestoquardi*, *ovis* or *parva*.

Our results reinforce LAMP as a test with a comparable sensitivity to classical molecular techniques (15, 19, 20) but more celerity and less requirement in terms of equipment and post-amplification manipulation than, other diagnostic tests (29–31). It also offers the advantage of using blood samples that can be easily obtained (32). Moreover, LAMP reaction is not affected by PCR inhibitors found in blood components (33–35). Additionally, the performance of this test can be improved as the pre-LAMP steps, sample processing, and DNA extraction can be optimized as well as the relative temperature stability of the LAMP reagents enabling the field deployment of the technique (17).

## Conclusion

The *T. annulata* species-specific LAMP assay described in the present work could be used to develop rapidly *in situ* molecular diagnostic tests which represent an important aspect of the early diagnosis and prompt treatment of theileriosis. Being able to detect and differentiate *Theileria annulata* with a technique that combines the reliability of molecular techniques together with the low cost and technical requirements, opens new horizons for production of high precision LAMP-based devices with improved sensitivity.
